# IgG deposition in IgA nephropathy patients

**DOI:** 10.12861/jrip.2013.06

**Published:** 2013-03-01

**Authors:** Hamid Nasri

**Affiliations:** ^1^Department of Nephrology, Division of Nephropathology, Isfahan University of Medical Sciences, Isfahan, Iran

**Keywords:** IgA nephropathy, Oxford classification, Immunostaining data

## Abstract

IgA nephropathy is the most common form of glomerular disease among young adults. The aim of this study is to determine the correlation of IgG deposition with morphologic variables of Oxford classification and some clinical data of patients with IgA nephropathy (IgAN).A total of 114 biopsies were enrolled to the study (70.2% were male). Mean age of the patients was 37.7±13.6 years. This study showed that, IgG deposition intensity had not significant correlation with serum creatinine. No significant association of sex with IgG was found. There was not significant association of IgG deposits with age below and more that 40 years. There was not significant association of IgG deposit intensity with four morphologic variables of Oxford classification. Less studied published regarding the immunostaining findings in IgA nephropathy patients. Location of deposited immunoglobulin (mesangial versus mesangial-capillary) or the type of immunoglobulin (IgG or IgM) may have prognostic significant. More studies needs to find the clinical significance of immunostaining data.

**Implication for health policy/practice/research/medical education:**

Less studied published regarding the immunostaining findings in IgA nephropathy patients. Location of deposited immunoglobulin (mesangial versus mesangial-capillary) or the type of immunoglobulin (IgG or IgM) may have prognostic significant. More studies needs to find the clinical significance of immunostaining data.

## Introduction

IgA nephropathy (IgAN) is the most common primary glomerular disease worldwide (1). IgAN is also characterized by mesangial deposits of IgA, often with In IgAN, there was also co-deposits of C3, IgG and IgAm with lower intensity in addition to IgA, and is a frequent finding. Recent studies showed that IgG deposition in IgAN or may have clinical significance (1,2). However, it is still unknown whether the immunostaining data are of prognostic significance. Studies concerning correlation of IgG deposition with morphologic findings of Oxford classification and clinical data are quite scarce (1-3). In this study we sought to investigate the association of immunostaining data with four morphologic variables of Oxford classification and also with various clinical data in a group of primary IgAN patients.

## Patients and Methods

### Pathologic definition of IgAN

The pathologic diagnosis of IgAN requires the demonstration of IgA-dominant mesangial or mesangio-capillary immune deposits through immunofluorescence (IF) microscopy. The immune deposits were semiquantitied from 0 to 3+ positive bright. The definition of IgAN needs the presence of diffuse and global IgA deposits that were graded ≥2+ and the absence of C_1_q deposition. Patients with systemic diseases such as diabetes mellitus, collagen diseases and chronic liver diseases were excluded from this study. All renal biopsies from July 2009 to July 2012 were sent to our renal pathology laboratory. None of the patients was treated before the biopsy. Biopsies with less than 8 glomeruli were also excluded from the study. IF slides were reported in a scales of 0 to 3+ positive bright. Data gathered at the time of biopsy were included race, gender, age, serum creatinine and proteinuria (based on a 24hour urine collection).

### Ethical issues

(1) The research followed the tenets of the Declaration of Helsinki; (2) informed consent was obtained; (3) the research was approved by the institutional review board.

### Statistical analysis

Mean values and standard deviations were calculated, and statistical signiﬁcance of the differences between groups were calculated using Mann-Whitney U-test and Chi-square tests. The Spearman’s coefficient of correlation was used to check the correlations. A computer program (SPSS version 17.0, Chicago, IL, USA) was used for statistical analysis. P< 0.05 was considered statistically significant.

## Results

This is an observational study, conducted on IgAN patients. A total of 114 biopsies were enrolled to the study. Mean age of patients was 37.7±13.6 years. IgG deposited intensity score was as follow; 74 patients had not deposition, one patient with 3+, 10 patients with 2+ and finally 29 patients with1+ intensity score. In this study, there was no correlation of IgG with IgA (p>0.05). There was no correlation of IgG with age or proteinuria (p>0.05). In this study, no significant association of IgG deposition with serum creatinine (p>0.05). In this study, no significant association of sex with IgG was found (p>0.05). There was no significant association of IgG deposit score with four morphologic variables of Oxford classification. Also no significant association of IgG deposition intensity with proportion crescents (p>0.05).

## Discussion

Less studied publish regarding the immunostaining findings in IgA nephropathy patients (3). In the study conducted by Bellur *et al*. on a group of IgAN patients, association of IgG staining with the presence of endocapillary proliferation and a higher mesangial cellularity score was found (4). This findings was not supported by our study. In the study of Bellur *et al*. the presence of IgG was not associated with the presence of focal and segmental glomerulosclerosis, crescents, interstitial or vascular lesions. Our study showed the same result too. They also found, no significant correlation of IgG deposits with urine protein excretion and glomerular filtration rate at the time of diagnosis. This finding is also in accord with our finding. In a recent study by Maeng *et al*. on 23 renal biopsy of IgAN, glomerular C4d deposition was associated with a higher grade of IgA nephropathy and higher amount of albuminuria (5). They concluded that activation of the complement system was involved in kidney damage and was identified through deposition of C4d in the glomeruli and tubules and positive C4d staining in the glomerulus and the tubules may be associated with functional damage related to glomerular filtration and poor kidney outcome. While, location of deposited immunoglobulin (mesangial *versus* mesangial-capillary) or the type of immunoglobulin (IgG or IgM) may have prognostic significant. More studies needs to find the clinical significance of immunostaining data.

## Author’s contribution

HN is the single author of the manuscript.

## Conflict of interests

The author declared no competing interests.

## Ethical considerations

Ethical issues (including plagiarism, data fabrication, double publication) have been completely observed by the author.

## Funding/Support

None.
